# Carbon Availability Modifies Temperature Responses of Heterotrophic Microbial Respiration, Carbon Uptake Affinity, and Stable Carbon Isotope Discrimination

**DOI:** 10.3389/fmicb.2016.02083

**Published:** 2016-12-26

**Authors:** Kyungjin Min, Christoph A. Lehmeier, Ford Ballantyne IV, Sharon A. Billings

**Affiliations:** Department of Ecology and Evolutionary Biology, Kansas Biological Survey, University of Kansas, LawrenceKS, USA

**Keywords:** carbon use efficiency, respiration, isotope, resource, microbial growth, chemostat, nutrient, stoichiometry

## Abstract

Microbial transformations of organic carbon (OC) generate a large flux of CO_2_ into the atmosphere and influence the C balance of terrestrial and aquatic ecosystems. Yet, inherent heterogeneity in natural environments precludes direct quantification of multiple microbial C fluxes that underlie CO_2_ production. Here we used a continuous flow bioreactor coupled with a stable C isotope analyzer to determine the effects of temperature and C availability (cellobiose concentration) on C fluxes and ^13^C discrimination of a microbial population growing at steady-state in a homogeneous, well-mixed environment. We estimated C uptake affinity and C use efficiency (CUE) to characterize the physiological responses of microbes to changing environmental conditions. Temperature increased biomass-C specific respiration rate and C uptake affinity at lower C availability, but did not influence those parameters at higher C availability. CUE decreased non-linearly with increasing temperature. The non-linear, negative relationship between CUE and temperature was more pronounced under lower C availability than under relatively high C availability. We observed stable isotope fractionation between C substrate and microbial biomass C (7~12‰ depletion), and between microbial biomass and respired CO_2_ (4~10‰ depletion). Microbial discrimination against ^13^C-containing cellobiose during C uptake was influenced by temperature and C availability, while discrimination during respiration was only influenced by C availability. Shifts in C uptake affinity with temperature and C availability may have modified uptake-induced ^13^C fractionation. By stressing the importance of C availability on temperature responses of microbial C fluxes, C uptake affinity, CUE, and isotopic fractionation, this study contributes to a fundamental understanding of C flow through microbes. This will help guide parameterization of microbial responses to varying temperature and C availability within Earth-system models.

## Introduction

Heterotrophic microorganisms break down organic carbon (OC) into assimilable molecules, which are subsequently incorporated into biomass or returned to the environment as exudates or respired CO_2_. In doing so, microbes influence the quantity and biochemical composition of OC in terrestrial and aquatic systems as well as atmospheric CO_2_ concentration. Because of the important role they play in regulating OC in terrestrial and aquatic systems and atmospheric CO_2_, ecosystem scientists have explicitly incorporated microbial dynamics into Earth system models (Allison et al., 2010; Wieder et al., 2013; Hagerty et al., 2014; Tang and Riley, 2015). However, it remains unclear what values should be used for microbial parameters [e.g., C use efficiency (CUE), biomass-C specific respiration] and how these parameters change with environmental conditions (Luo et al., 2016). This is because heterogeneity in natural environments, especially in soils, makes it nearly impossible to empirically determine many of these parameters *in situ* (Werth and Kuzyakov, 2010; Billings and Ballantyne, 2013; Billings et al., 2015).

Assessing microbial OC transformations in controlled, experimental systems can help us overcome some of the difficulties ecosystem scientists face when using natural samples to investigate microbial C flows (Billings et al., 2015). For example, chemostats, continuous flow bioreactors for the cultivation of microorganisms, have been widely employed to investigate fundamental microbial physiology during OC transformations by removing the influence of natural heterogeneity (Rhee and Gotham, 1981; Cajal-Medrano and Maske, 2005; Cotner et al., 2006; Chrzanowski and Grover, 2008; Ferenci, 2008). Recently, Lehmeier et al. (2016) used a coupled chemostat and C isotope analyzer to improve our understanding of C flows from substrate through microbial biomass and into respired CO_2_, which is of great interest at multiple scales and levels of organization, from microbes to ecosystems (Blair et al., 1985; Hagström et al., 1988; Henn and Chapela, 2000; Šantrůčková et al., 2000; Henn et al., 2002; Werth and Kuzyakov, 2010; Brüggemann et al., 2011; Dijkstra et al., 2011a,b; Penger et al., 2014). In particular, quantifying the effects of temperature on biomass-C specific respiration rate, CUE, and microbial ^13^C discrimination for a ubiquitous microbe (Lehmeier et al., 2016) provides a mechanistic basis and justification for the often assumed temperature dependence of CUE in Earth-system models.

Temperature responses of microbial OC transformations in Lehmeier et al. (2016) were quantified using a single C substrate, cellobiose, at a single concentration, 10 mM. However, it remains unknown how the temperature responses of microbial respiration, CUE, and ^13^C discrimination may be influenced by C availability which varies among ecosystems, vertically and laterally throughout soil profiles, and as microbes’ substrate landscapes shift over time (Kuzyakov, 2010; Richter and Markewitz, 2013). Furthermore, the absolute and relative abundance of readily assimilable C can also vary with temperature (Bárta et al., 2013; Lehmeier et al., 2013; Min et al., 2014), and thus can influence the temperature responses of microbial OC transformations (Rhee and Gotham, 1981; Pomeroy and Wiebe, 2001; Hall and Cotner, 2007; Manzoni et al., 2012; Billings and Ballantyne, 2013; Frey et al., 2013; Xu et al., 2014).

When C availability decreases, microorganisms can adjust their ability to take up C as a compensatory measure (Death et al., 1993; Williams et al., 1994; Ferenci, 1999; Gresham and Hong, 2015). The ability to take up C, or C uptake affinity, is often defined as the ratio of the maximum uptake rate (*V_max_*) to the half-saturation concentration of C (*k_m_*) of the Michaelis-Menten function (Healey, 1980; Aksnes and Egge, 1991; Button, 1993; Reay et al., 1999). Because C uptake affinity defined this way is equivalent to the slope of the Michaelis–Menten function at a C concentration of zero, it reflects the sensitivity of the uptake response to changing C availability at low absolute concentrations. This suggests that microbes may allocate resources to a more energetically costly, high affinity C uptake strategy at relatively low C availability (Patzer and Hantke, 1998), resulting in enhanced respiratory costs. Furthermore, changing C uptake affinity may alter ^13^C discrimination during uptake and subsequently influence δ^13^C of remaining OC pools, as well as downstream pools such as biomass and respired CO_2_ (Lehmeier et al., 2016). Understanding any such C uptake affinity-induced shift in fractionation would be critical to correctly interpret δ^13^C of terrestrial C pools and fluxes.

We grew *Pseudomonas fluorescens*, a cosmopolitan Gram-negative bacterium, in a chemostat system coupled to a ^13^CO_2_/^12^CO_2_ analyzer and quantified how the sizes of C pools in the system and their C stable isotope ratios varied with temperature and C availability. Combining the measurements with a dynamic model of substrate-C and biomass-C, we estimated how C uptake and C uptake affinity varied with changing temperature (11.5–25.5°C) and C availability (1 and 20 mM cellobiose). The cellobiose concentrations of 1 and 20 mM yielded medium C:N of 1 and 20, respectively, to span the observed range of C:N of bacterial biomass (5–10) reported in Nagata (1986) and Cleveland and Liptzin (2007). Our primary goal was to quantify how changing C substrate availability alters the temperature dependence of C flow through a common microbe and associated C stable isotope fractionations. As such, we asked: (1) how will temperature and C availability influence biomass-C specific respiration, C uptake affinity, and CUE during OC transformations? (2) how will changes in these microbial parameters with temperature and C availability, if any, modify ^13^C discrimination among substrate, biomass and respired CO_2_?

## Materials and Methods

The chemostat system coupled to a continuous flow ^13^CO_2_/^12^CO_2_ gas analyzer employed in this study is described in full detail in Lehmeier et al. (2016). Here we summarize the protocol of running a chemostat experiment and expand Lehmeier et al.’s approach by providing estimates for C uptake affinity and a simple model for CUE.

### Microorganisms and Nutrient Solution With Two Contrasting C Concentrations

We used *Pseudomonas fluorescens* (ATCC ^®^13525, Carolina Biological Supply, USA), because it is one of the most common bacteria inhabiting both soil and water. As growth medium for *P. fluorescens*, we prepared nutrient solution modified from Abraham et al. (1998), containing 10 mM NH_4_Cl, 1.6 mM KNO_3_, 2.6 mM K_2_HPO_4_, 1.0 mM KH_2_PO_4_, 0.8 mM MgSO_4_, 0.2 mM CaCl_2_, 0.1 mM CuCl_2_, 0.04 mM FeSO_4_, 0.03 mM MnCl_2_ and 0.02 mM ZnSO_4_. After adjustment to pH 6.5, the nutrient solution was autoclaved and stored under UV light. We added cellobiose (Sigma–Aldrich, USA) to the sterile nutrient solution as the only organic C source, inoculated it with *P. fluorescens*, and grew cultures at 10°C. These pre-cultures of *P. fluorescens* served as inoculum for the chemostat bioreactor. The concentration of cellobiose in the nutrient solution was adjusted to either 1 or 20 mM, which corresponded to resource C to N ratios of 1 and 20, respectively. Cellobiose δ^13^C values ranged from –24.4 to –24.9‰, depending on the lot from which the supply was issued. For each individual chemostat run, we used cellobiose derived from only one lot to ensure a constant δ^13^C value of microbial substrate for each run (Supplementary Table 1).

### Continuous Flow Chemostat System

The chemostat system consisted of two 1.9 L Mason jars (Ball, USA), which were connected via flexible tubing (Masterflex Tygon E-LFL tubing, Cole-Parmer, USA) and situated on stir plates in separate incubators. One jar functioned as a bioreactor, into which we poured 1 L of fresh nutrient solution and injected inoculum (5 mL of *P. fluorescens* pre-culture) at the start of each individual chemostat run; the other jar functioned as a reservoir for fresh nutrient solution and was regularly refilled during a chemostat run.

**FIGURE 1:**
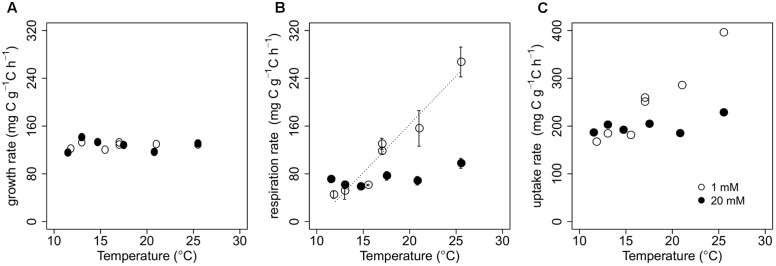
**Biomass-C specific rates of growth (A)**, respiration **(B)**, and uptake **(C)** with varying temperature for *P. fluorescens* grown in chemostats with cellobiose concentrations in the nutrient solution of either 1 mM (open circles) or 20 mM (closed circles). We imposed a regression line on data points when there was a significant temperature effect on rates (see Supplementary Table 2). Error bars in (B) represent ± 1SE.

In the continuous culture mode, a peristaltic pump supplied fresh nutrient solution from the reservoir to the bioreactor at a constant rate, providing new resources to the growing microbial population. The pump simultaneously removed well-mixed bioreactor medium (including microbial cells) at the same rate as the nutrient solution supply rate via a waste line, such that the total volume of liquid in the bioreactor remained constant. The dilution rate of the chemostat bioreactor averaged 0.128 h^–1^ (**Figure 1A**). By definition, the dilution rate equals the growth rate of the microbial population in the chemostat reactor under steady-state conditions (Hoskisson and Hobbs, 2005; Bull, 2010). Steady state in this sense means that the microbial population in the bioreactor reaches a state of constant size and activity under the prevailing environmental conditions, and that the number of cells leaving the bioreactor via dilution is counterbalanced by cell division.

Analogous to the continuous flow of liquid, gas also flowed continuously through the bioreactor. A ^13^CO_2_/^12^CO_2_ analyzer (G2101-i, Picarro, USA) removed headspace air from the bioreactor at a rate of 25 mL min^–1^ on average and continuously measured the concentration and the δ^13^C of headspace CO_2_. The air volume removed was instantaneously replaced with CO_2_-free air, bubbled into the nutrient solution at the same rate that the ^13^CO_2_/^12^CO_2_ analyzer withdrew headspace air via a mass flow controller (MC-50SCCM, Alicat Scientific, USA). In this way, bioreactor headspace air pressure remained constant and the CO_2_-free air provided the microbial population with oxygen.

Immediately after the inoculation and closure of the chemostat bioreactor, CO_2_ concentration in the headspace air represented lab air conditions (~400 ppm CO_2_). Soon after closure of the bioreactor, the CO_2_ concentration approached zero because of the bubbling of CO_2_-free air into the bioreactor and little microbial activity. Subsequently, the size of the microbial population and hence the rate of respiratory CO_2_ production increased, and after the switch to the continuous culture mode, it achieved steady state conditions. Prior to the steady state, the ^13^CO_2_/^12^CO_2_ measurement of the headspace air did not solely reflect microbial respiration, because the bioreactor medium acted as a sink for respired CO_2_ (in the form of H_2_CO_3_ and ${\text{HCO}}_{3}^{-}$; Stumm and Morgan, 1981; Lehmeier et al., 2016). When the microbial population reached steady state, both the CO_2_ production rate and the δ^13^C of the CO_2_ became constant, indicating that chemical and isotopic equilibria of the carbonate system were achieved. However, even in the steady-state phase, not all of the respired CO_2_ entering the bioreactor medium was released into the bioreactor headspace. This is because some respired CO_2_ entered the carbonate system and was removed via the waste line due to dilution. We estimated that not more than 6~8% of respired CO_2_ was lost from the bioreactor via dilution, hence, we may have underestimated microbial respiration rates by about 6–8%. Importantly, as both the supply rates of fresh medium and the steady-state CO_2_ concentrations in the bioreactor headspace (the two most important factors determining the sink strength of the supplied fresh medium) were very similar across all chemostat runs, the degree of underestimating microbial respiration rate was very similar across chemostat runs. Therefore, we considered the headspace CO_2_ representing the concentration and δ^13^C of respired CO_2_ (for more detailed information, please refer to the Supplementary Material in Lehmeier et al., 2016). We thus used measurements of bioreactor headspace air when the microbial population was at steady state to quantify microbial respiration rates and δ^13^C of respired CO_2_, allowing us to draw conclusions about microbial behavior without concern for interactions between headspace CO_2_ and inorganic C pools in the bioreactor medium.

**FIGURE 2:**
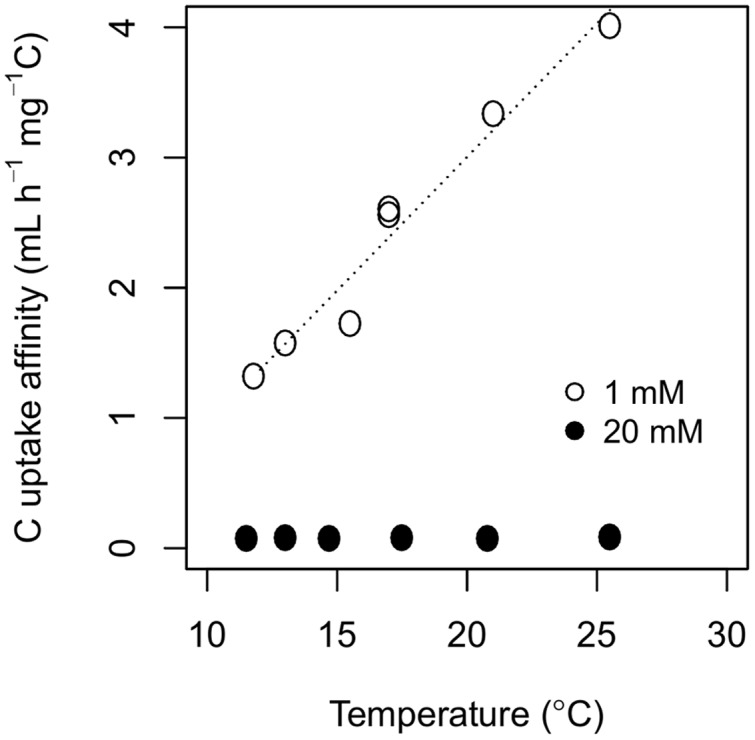
**Temperature responses of C uptake affinity, the slope of *V_max_* over *k_m_* (see Estimates of C Uptake Affinity and CUE), estimated from the two-pool model of *P. fluorescens* grown in chemostats with cellobiose concentrations in the nutrient solution of either 1 mM (open circles) or 20 mM (closed circles).** Model parameters are available in Supplementary Table 2.

**FIGURE 3:**
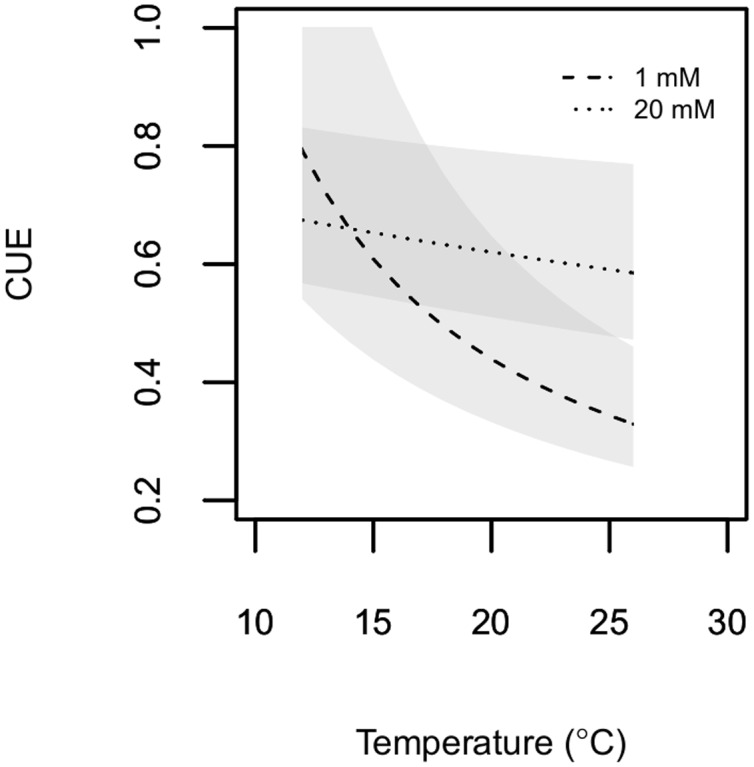
**Estimated microbial CUE is plotted as a function of temperature.** *P. fluorescens* was grown in chemostats with cellobiose concentrations in the nutrient solution of either1 mM (dashed line) or 20 mM (dotted line). Shaded areas represent 95% confidence envelopes of estimated CUE.

**FIGURE 4:**
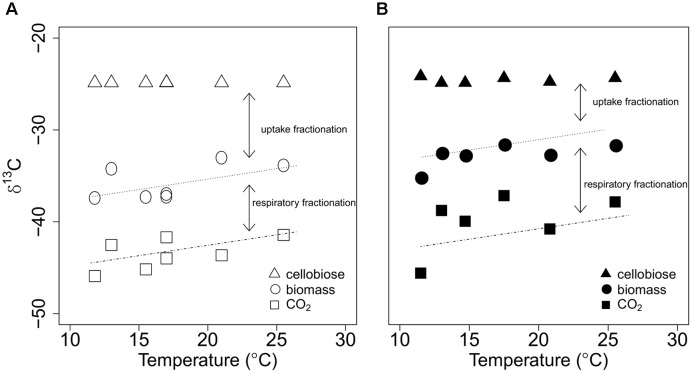
**δ^13^C values of provided cellobiose (the sole OC source in the system; triangles), and of microbial biomass (circles), and respired CO_2_ (squares) of *P. fluorescens* in chemostats with cellobiose concentrations in the nutrient solution of either 1 mM (A)** or 20 mM **(B)** are plotted against bioreactor temperature. Regression lines represent statistically significant, common temperature effects on δ^13^C of microbial biomass and respired CO_2_ (see Supplementary Table 2). As indicated by arrows, uptake fractionation is the difference in δ^13^C values between cellobiose and biomass, and respiratory fractionation is the difference in δ^13^C values between biomass and respired CO_2._

For each cellobiose concentration, chemostat experiments were conducted at six different bioreactor temperatures, randomly ordered: at 1 mM of cellobiose, reactor temperature was maintained at 11.8, 13, 15.5, 17, 21, or 25.5°C; at 20 mM of cellobiose, it was 11.5, 13, 14.7, 17.5, 20.8, or 25.5°C. This experimental temperature range covers an ecologically relevant range of temperatures in many ecosystems. Below 11.5°C, microorganisms’ steady-state growth was unsustainable at the dilution rate of 0.128 h^–1^. This is consistent with findings in Höfle (1979), which reported that the maximum growth rate of *P. fluorescens* at 10°C is 0.13 h^–1^. We conducted one repeated chemostat run at 17°C and 1 mM of cellobiose. We acknowledge that one replication is not enough to quantify an overall uncertainty of the measurements, but similar values of microbial OC transformations for these replicated runs, in conjunction with clear temperature-related trends (see Results), suggest that our measurements were reliable (**Figures 1**–**4**).

### Quantification of Microbial Biomass and Respiration at Steady-State

At steady state, we collected and filtered the bioreactor medium from the waste line on dry, pre-weighed 0.22 μm polyethersulfone filters (Pall, USA). We dried the wet filters at 75°C for 48 h in an oven and reweighed them to determine dry microbial biomass. Carbon and N in microbial biomass were quantified using an elemental analyzer coupled to an isotope-ratio mass spectrometer, either at the Keck Paleoenvironmental and Environmental Stable Isotope Laboratory at the University of Kansas or at the Stable Isotope Laboratory at the University of Arkansas. In both laboratories, the standard deviation of repeated lab standard measurements was a maximum of 0.2‰.

We calculated microbial respiration rates by multiplying the average reactor headspace CO_2_ concentration measured over 5 h at steady state with the molar air flow rate through the bioreactor. After steady state CO_2_ measurements for each chemostat run, we measured a laboratory standard gas, which had a CO_2_ concentration of 1015 ppm and a δ^13^C of –48.9‰ as calibrated against secondary CO_2_ standards from KU’s Keck Paleoenvironmental and Environmental Stable Isotope Laboratory. This served to check the concentrations and to correct the isotopic signatures of bioreactor headspace CO_2_ measurements.

### Carbon Dynamics in the Chemostat Bioreactor

In a chemostat bioreactor provided with cellobiose as the sole OC substrate there are two OC pools, cellobiose-C and biomass-C, if we assume exudation is negligible (Blair et al., 1985; Hagström et al., 1988). The temporal dynamics of the two OC pools can be described with two ordinary differential equations (Smith and Waltman, 1995).

(1) $$\begin{array}{cc} \frac{dS}{dt}=(I-S)d-Bf\left(S\right) & \end{array}$$

(2) $$\begin{array}{cc} \frac{dB}{dt}=B\left(d(S)-\left(d+\rho +ɛ\right)\right) & \end{array}$$

where *S* is cellobiose-C in the chemostat bioreactor, *I* is cellobiose-C input to the chemostat bioreactor, *d* is the dilution rate (which equals the biomass-C-specific population growth rate in a steady-state chemostat; Hoskisson and Hobbs, 2005; Bull, 2010), *B* is biomass-C in the chemostat reactor, *f(S)* is the function describing biomass-C specific uptake rate of cellobiose-C, *ρ* is the biomass-C specific respiration rate, 𝜀 is the biomass-C specific exudation rate, and *t* is time. At steady state, denoted by ^∧^, biomass-C and cellobiose-C concentrations are both constant $\left(\frac{dB}{dt}=\frac{dS}{dt}=0\right)$. This means that the biomass-C specific uptake rate, *f(S)*, equals the sum of *d*, *ρ* and *𝜀*. If we assume *𝜀* is negligible, *f(S)* is estimated as the sum of *d* and *ρ*. The steady state conditions in the chemostat allow biomass-C specific rates to be computed by dividing gross C fluxes by C pool sizes. For example, *ρ* was estimated by dividing the total rate of respiration of the bioreactor by microbial biomass-C at steady state, which we obtained via elemental analysis of dried biomass.

### Estimates of C Uptake Affinity and CUE

Although the steady state measurements of *d* and *ρ* allow us to estimate *f(S)*, this alone does not provide the physiological basis for a change in uptake fluxes. However, if we employ a standard assumption of *f(S)* saturating as a function of C availability (Button, 1993; Ferenci, 1999), we can begin to probe temperature-dependent physiological shifts. We can express *f(S)*, the function describing the biomass-C specific uptake rate of cellobiose-C (see equation 1), with the standard Michaelis-Menten formulation for cellobiose-C uptake as

(3) $$\begin{array}{cc} f\left(S\right)=\frac{V_{\max}\;S}{S+k_{m}} & \end{array}$$

This approach allows us to relate changes in C uptake physiology, namely in *V_max_* and *k_m_*, to changing temperature, such that a change in C uptake physiology would be reflected in a change in either or both with temperature and C availability.

Assuming that *𝜀* is negligible and combining equations 2 and 3, we can express steady-state concentrations of biomass as

(4) B^=(1−km(d+ρ)Vmax−d−ρ)*dd+ρ

We can rearrange equation 4 to consolidate all our measurements and pre-specified quantities on the right hand side:

(5) kmVmax−d−ρ=Id+ρ−B^d

For convenience, we replace the right side of equation 5 with *A* and rearrange terms to arrive at

(6) $$\begin{array}{cc} V_{max}=\frac{k_{m}}{A}+d+\rho & \end{array}$$

Here, *1/A* represents the slope of *V_max_* over *k_m_* and equals $\frac{V_{\max}-d-\rho }{k_{m}}$. If we assume *V_max_* is greatly larger than the sum of *d* and *ρ* (Schulze and Lipe, 1964; Button, 1985; Kovárová-Kovar and Egli, 1998), then *1/A* approaches C uptake affinity, defined as the ratio of *V_max_* over *k_m_* (Button, 1993; Nedwell, 1999; Reay et al., 1999). Thus, hereafter we refer to *1/A* as estimated cellobiose-C uptake affinity for *P. fluorescens* at steady state. Our estimates of C uptake affinity are valuable because they provide insight to how *P. fluorescens* responds physiologically to changing temperature and C availability, without independently quantifying the kinetic parameters *V_max_* and *k_m_*.

We estimated CUE by substituting the apparently linear relationship between biomass-C specific respiration rate and temperature (see Statistics below) via equation 7:

(7) $$\begin{array}{cc} {\begin{array}{c} CUE \end{array}}_{i}=\frac{d}{d+\rho }=\frac{d}{d+\left(\alpha_{i}+\beta_{i}*T\right)} & \end{array}$$

where *i* corresponds to either 1 or 20 mM cellobiose concentrations in the nutrient medium, *α_i_* is the y-intercept of the regression line relating biomass-C specific respiration rate to temperature at each C availability, and *β_i_* is the slope of the line at each C availability. Using errors obtained from the modeled temperature-dependence of biomass-C specific respiration rates, we calculated 95% confidence envelopes surrounding these CUE estimates. Because the dilution rate of the chemostat bioreactor was relatively constant at 0.128 h^–1^, changes in CUE estimates were driven by temperature-dependent changes in biomass-C specific respiration rates.

### Statistics

We used general linear models to relate biomass-C specific respiration rate and C uptake affinity to temperature and cellobiose concentration in the nutrient solution. Both models consider temperature as a continuous covariate, and cellobiose concentration as a categorical predictor. Because biomass-C specific respiration rates were a key feature of our estimates of biomass-C specific uptake rates and CUE (see Carbon Dynamics in the Chemostat Bioreactor), we did not fit separate models for these two responses to test the effects of temperature and cellobiose concentration. For the C stable isotope data, we treated temperature as a continuous covariate, and cellobiose concentration and C pool (biomass and CO_2_) as categorical predictors. To correct any effect of varying δ^13^C of cellobiose across experiments on δ^13^C of both biomass and CO_2_, we used the differences between δ^13^C of cellobiose and δ^13^C of biomass, and between δ^13^C of cellobiose and δ^13^C of CO_2_, as dependent variables. We used the Akaike Information Criterion (AIC) to inform model selection for each of the three response variables. All statistical analyses were performed with R (R version 3.1.2. R development core team).

## Results

### Microbial Biomass-C Specific Rates: Respiration, Growth, and Uptake

Increasing temperature significantly enhanced biomass-C specific respiration at 1 mM of cellobiose (*p* < 0.001), while temperature did not significantly influence biomass-C specific respiration at 20 mM of cellobiose (**Figure 1B**; Supplementary Table 2). Because our estimates of biomass-C specific uptake were computed by summing biomass-C specific rates of growth and respiration (see Carbon Dynamics in the Chemostat Bioreactor), and biomass-C specific growth rate was relatively constant across all chemostat runs (**Figure 1A**), temperature responses of biomass-C specific uptake rates were similar to those of biomass-C specific respiration at each C availability (**Figure 1C**). Biomass-C specific uptake rates at both C availabilities were similar at lower temperatures, but diverged with increasing temperature. At 25.5°C, biomass-C specific uptake rate of the microbes at 1 mM cellobiose was almost twice as high as the biomass-C specific uptake rates at 20 mM cellobiose. We calculated that *P. fluorescens* took up, on average across temperatures, 17.4 ± 1.9 and 1.3 ± 0.1% of the cellobiose-C substrate supplied per hour at 1 and 20 mM, respectively.

### Estimates of C Uptake Affinity and CUE

The interactive effect of temperature and C availability on biomass-C specific respiration rate influenced the estimates of C uptake affinity and CUE. As temperature increased from 11.5 to 25.5°C, C uptake affinity increased from 1.31 to 4.01 mL h^–1^ mg^–1^ C at 1 mM of cellobiose, but temperature did not influence C uptake affinity at 20 mM of cellobiose (**Figure 2**; Supplementary Table 2). Carbon uptake affinity was always higher at 1 mM of cellobiose than at 20 mM, and exhibited no convergence at the lowest temperature in contrast to the biomass-C specific uptake and respiration rates.

Microbial CUE ranged between 0.3 and 0.8, with similar maximum values for both C availabilities (**Figure 3**). Within the experimental temperature range, microbial CUE decreased non-linearly with increasing temperature. The non-linear nature of the relationship between temperature and CUE estimates (see equation 7) was much more pronounced at 1 mM cellobiose than at 20 mM cellobiose. This was a consequence of the much greater temperature response of biomass-C specific respiration and of the associated difference between biomass-C specific respiration and growth rates at 1 mM cellobiose.

### δ^13^C of C Pools and Isotopic Fractionations

Temperature and C availability independently influenced δ^13^C of microbial biomass and respired CO_2_ (**Table 1**; Supplementary Table 2). When cellobiose was incorporated into microbial biomass and eventually respired as CO_2_, large fractionations were apparent at both 1 and 20 mM cellobiose (**Figure 4**). The value of δ^13^C for biomass was always more negative than δ^13^C of the cellobiose, and δ^13^C of respired CO_2_ was even more negative than δ^13^C of the biomass at both C availabilities. The degree to which temperature increased both δ^13^C of biomass and respired CO_2_ was statistically indistinguishable for both cellobiose concentrations, as illustrated by the common slope coefficient depicted in **Figure 4** (slope = 0.23‰ increase in δ^13^C per °C, p = 0.007). The values of δ^13^C of biomass and respired CO_2_ were always more negative at 1 mM cellobiose than at 20 mM cellobiose. Assuming that *P. fluorescens* did not exude significant amounts of C, standard isotopic mass balance dictates that the δ^13^C of C taken up by the bacteria must have been more negative than the δ^13^C of the cellobiose substrate (Supplementary Table 1). This apparent discrimination against ^13^C-containing cellobiose molecules during uptake was influenced by temperature and C availability. More specifically, the lower the C availability and the lower the temperature, the greater the fractionation during uptake (the differences in δ^13^C of cellobiose and δ^13^C of microbial biomass in **Figure 4**). In contrast, fractionation during respiration (the differences in δ^13^C of microbial biomass and δ^13^C of respired CO_2_) did not vary significantly with temperature, as depicted by parallel regression lines in **Figure 4**.

**Table 1:** Analysis of covariance results testing the effects of microbial C pools, temperature, and C availability on δ^13^C of cellobiose, biomass, and respired CO_2_ during cellobiose transformations.

	df	SS	MS	*F*-value	*P*-value
C pool	1	365.11	365.11	111.6171	**<0.001**
Temperature	1	27.26	27.26	8.3337	** 0.007**
C availability	1	54.77	54.77	16.7443	** <0.001**
residuals	22	66.05	3.00		

*Microbial C pools represent biomass and respired CO_2_. Significant effects are highlighted in bold.*

## Discussion

We used chemostats and stable C isotope analyses to quantify rates and isotopic signatures of C flowing from a single C substrate through microbial biomass into CO_2_. Using a two C pool dynamic model for substrate and biomass coupled with measurements of pool sizes and fluxes, we estimated how microbial C uptake, C uptake affinity, and CUE change with temperature and C availability. The combination of modeling and measurements allowed us to infer changes with environmental conditions in underlying microbial physiology. Our results highlight how temperature and C availability influence biomass-C specific rates and microbial ^13^C discrimination during OC transformations. Below, we discuss the inferred, underlying mechanistic basis for the observed responses to varying temperature and C availability, and implications for interpreting the isotopic signature of ecosystem respiration.

### Interactive Effects of Temperature and C Availability on C Uptake Affinity, Biomass-C Specific Rates, and CUE

We compared microbial C fluxes at 1 and 20 mM cellobiose (current study) with those at 10 mM cellobiose as reported in Lehmeier et al. (2016). Microbial biomass-C specific rates, C uptake affinity, and biomass-C specific C uptake rates at 10 mM cellobiose fall between those reported here at 1 and 20 mM cellobiose, suggesting gradual changes in microbial temperature responses to C availability. The shifts in C uptake affinity and C uptake rates from 1 mM to 10 mM to 20 mM are consistent with the notion that microorganisms can sense their environment and adjust their ability to acquire C as substrate availability varies (Williams et al., 1994; Ferenci, 1999; Gresham and Hong, 2015). Indeed, microorganisms possess multiple C transporter systems, each exhibiting varying affinity, and these systems’ relative expression can change with environmental C concentrations (Williams et al., 1994; Schlösser and Schrempf, 1996; Ferenci, 1999; Kajikawa and Masaki, 1999; Koning et al., 2001; Gresham and Hong, 2015). It is beyond the scope of our study to investigate the exact mechanisms driving shifts in C uptake affinity. However, enhanced C uptake affinity can occur through various mechanisms, including changes in transporter density, microbial surface to volume ratio, membrane thickness, and gene expression of a suite of transporters with different affinity (Aksnes and Egge, 1991; Button, 1993; Kovárová-Kovar and Egli, 1998; Liu and Ferenci, 2001; Gresham and Hong, 2015).

Our system is different from natural systems in that one population of microorganisms grew with surplus C at steady state without interactions with other microbial populations. This distinction allows us to make multiple observations about microbial C flows with only temperature and C availability varying, which cannot be addressed directly using environmental samples. First, our work reinforces the idea that CUE declines with rising temperature (Steinweg et al., 2008; Allison et al., 2010; Tucker et al., 2013; Wieder et al., 2013; Schindlbacher et al., 2015), though this has not always been inferred in studies using environmental samples (Crowther and Bradford, 2013; Hagerty et al., 2014). The maximum, convergent CUE around 0.7-0.8 in the current study and in Lehmeier et al. (2016) is consistent with inferred CUE values reported in aquatic systems (Hall and Cotner, 2007; Manzoni et al., 2012), soils (Dijkstra et al., 2011b; Manzoni et al., 2012; Frey et al., 2013; Blagodatskaya et al., 2014; Lee and Schmidt, 2014), and batch culture (Keiblinger et al., 2010). The ceiling value of CUE suggests that inherent limitations might keep microorganisms from achieving higher CUE values. At relatively low temperatures, microbial respiratory costs for growth, maintenance, and foraging are minimized (Rivkin and Legendre, 2001; Apple et al., 2006), likely yielding relatively high CUE. However, we expect that the negative relationship between CUE and temperature breaks down below a certain temperature limit: when microbes enter dormancy at extremely low temperature (i.e., growth rate is null), then CUE defined as growth rate/(growth rate + respiration rate) approaches zero. Thus, we highlight that current conceptions and estimates of CUE are well-defined only when C is flowing into microbial cells and that the negative CUE-temperature relationship may only hold true at temperatures at which microbes are growing.

Second, our results demonstrate that assuming a linear decline of CUE with temperature (Allison et al., 2010; Wieder et al., 2013) may not be appropriate. Indeed, CUE as defined here and in multiple other studies (see Manzoni et al., 2012) must decline with temperature in a non-linear fashion if respiration increases in a linear fashion as observed in this (**Figure 1B**) and a related study (Lehmeier et al., 2016). Our observation of a non-linear temperature dependence of CUE estimates emphasizes the need for further research to advance CUE estimates in Earth system models (Ballantyne and Billings in review).

Third, greater temperature responses of respiration and CUE at relatively low C availability, as observed in this study, support the idea that lower resource C:N may be generally linked to higher temperature sensitivity of OC transformations (Fierer et al., 2003; Rumpel and Kögel-Knabner, 2011; Billings and Ballantyne, 2013). We acknowledge that the meaning of resource C:N in our chemostat system (as determined by the ratio of cellobiose-C to mineral N) may be different from that in natural conditions, where microbes have varying access to both organic and inorganic mineral nutrients and resources can be protected physically and chemically. Furthermore, we do not know if changes in C availability caused nutrient limitation or a shift in relative nutrient limitation, common in natural conditions. However, if these results are robust and are not masked by the myriad other features at play in natural systems, we would predict that environments exhibiting relatively lower C concentrations such as deep, relatively old soil OC and fertilized soils/aquatic systems would be especially vulnerable to OC losses via microbial respiration with warming.

### Temperature and C Availability Effects on Microbial C Isotope Discrimination

Our isotope data suggest that C availability can significantly alter microbial uptake as well as uptake discrimination against ^13^C-containing compounds. Bigger δ^13^C differences between cellobiose and microbial biomass at low C availability can be partly explained by greater C uptake affinity and associated biomass-C specific uptake rates. Shifts from a low C affinity uptake system to a high C uptake affinity system at lower C availability may lead to greater uptake fractionation, given that transport pathways with different affinities can exhibit varying degrees of C isotope fractionation (Henn and Chapela, 2000). However, even if different C uptake affinity systems have the same isotopic fractionation effect, greater uptake flux rates through a higher C uptake affinity system can enhance the magnitude of ^13^C discrimination during protein-mediated reactions (Tcherkez et al., 2011). Although these scenarios provide potential clues explaining why stable C uptake fractionation was higher at low C availability, they cannot explain why the degree of microbial ^13^C discrimination during uptake apparently was not influenced by changes in temperature (**Figure 4**). Thus, further studies need to examine the degree of coupling between changing C uptake affinity/uptake rate and uptake ^13^C fractionation under different environmental conditions.

Contrary to Lehmeier et al. (2016), we observed similar respiratory fractionation across temperatures for both cellobiose concentrations, as illustrated by two parallel regression lines in **Figure 4**. If we assume that this similar respiratory fractionation can be used to interpret δ^13^C values of microbial biomass and respired CO_2_ in natural samples, our results may help tease apart the effects of multiple, confounding factors driving apparent respiratory C isotope fractionation in environmental samples. In natural samples, respiratory fractionation is a net result of kinetic isotope effects, preferential substrate utilization, and heterogeneity in microbial community composition (Werth and Kuzyakov, 2010). By controlling the type and supply of substrate, and employing one population of microorganisms, our results approach intrinsic kinetic isotope effects as much as possible, with little influence of other factors. Thus, comparison of respiratory fractionation in this study to that in natural samples can be useful for quantifying the magnitude and direction of the confounding factors influencing C isotope fractionations during microbial C mineralization. Direct comparisons should be made with caution, because the range of respiratory ^13^C discrimination in this study (from ~5 to ~11‰, the difference between δ^13^C of biomass and respired CO_2_) is higher than apparent respiratory ^13^C discrimination reported in other studies of natural samples (Šantrůčková et al., 2000; Werth and Kuzyakov, 2010; Brüggemann et al., 2011). This discrepancy may arise from the relatively high C availability and decoupled C and N sources in our chemostat system. High absolute C availability in chemostats may induce microorganisms to enhance discrimination against ^13^C during OC transformations, given reduced discrimination against heavy stable isotopes when resources are in relatively scant supply (Fry, 2006). However, our work provides baseline data describing the potential degree of discrimination against ^13^C during microbially induced C flows and how it may or may not change with environmental conditions.

Ecosystem scientists frequently assume that heterotrophic microbial ^13^C discrimination during OC transformations is minimal and that the δ^13^C value of soil-respired CO_2_ closely reflects the δ^13^C of the C source (Ehleringer et al., 2000; Bowling et al., 2008). This assumption may be valid for some scenarios. However, the results reported here demonstrate that microorganisms can discriminate substantially against ^13^C during uptake and respiration, and that the degree of discrimination during OC uptake may change with C availability and temperature. This is relevant for interpretations of δ^13^C of OC substrates in natural environments, microorganisms themselves (their live biomass, and their necromass’ contribution to OC), and respired CO_2_. These data serve as a cautionary tale for those using δ^13^C signatures to infer underlying mechanisms driving those values, or those projecting ecosystem C pools and fluxes.

## Author Contributions

KM, CL, FB, and SB contributed to the development of the idea. KM and CL performed the experiment. KM, FB, and SB analyzed the data and all were involved in the data interpretation. KM wrote a draft and all contributed to revising it.

## Conflict of Interest Statement

The authors declare that the research was conducted in the absence of any commercial or financial relationships that could be construed as a potential conflict of interest.
