# Supplementary material for: Carbon Availability Modifies Temperature Responses of Heterotrophic Microbial Respiration, Carbon Uptake Affinity, and Stable Carbon Isotope Discrimination
Source: Front Microbiol. 2016 Dec 26;7:2083. doi: 10.3389/fmicb.2016.02083 (PMC5184216; doi:10.3389/fmicb.2016.02083)
Supplement: Supplementary file 1 [file Table_1.docx]

Supplementary Table 1. δ^13^C of cellobiose provided to microbes, biomass, respired CO_2_, and C taken up into microbial cells in chemostat experiments with varying temperature and C availability. The values of δ^13^C for cellobiose, biomass and respired CO_2_ were directly quantified in our system (Figure 4), while δ^13^C of C taken up into microbial cells was estimated using a standard mixing model (C uptake rate * δ^13^C of C taken-up = C growth rate * δ^13^C of biomass + C respiration rate * δ^13^C of respired CO_2_), assuming that exudation of organic compounds by microbial cells was negligible.

| Temperature  (°C) | Cellobiose concentration (mM) | δ^13^C of cellobiose  (‰) | δ^13^C of biomass  (‰) | δ^13^C of respired CO_2_  (‰) | Estimated δ^13^C of C taken into cells  (‰) |
| --- | --- | --- | --- | --- | --- |
| 11.8 | 1 | -24.9 | -37.4 | -45.9 | -39.7 |
| 13 | 1 | -24.9 | -34.3 | -42.5 | -36.6 |
| 15.5 | 1 | -24.9 | -37.3 | -45.2 | -40.0 |
| 17 | 1 | -24.9 | -37.3 | -44.0 | -40.4 |
| 17 | 1 | -24.8 | -37.0 | -41.7 | -39.4 |
| 21 | 1 | -24.9 | -33.0 | -43.7 | -38.8 |
| 25.5 | 1 | -24.9 | -33.9 | -41.4 | -39.0 |
| 11.5 | 20 | -24.9 | -37.4 | -45.9 | -39.7 |
| 13 | 20 | -24.9 | -32.6 | -38.8 | -34.5 |
| 14.7 | 20 | -24.9 | -32.8 | -39.9 | -35.0 |
| 17.5 | 20 | -24.4 | -31.7 | -37.2 | -33.7 |
| 20.8 | 20 | -24.8 | -32.8 | -40.8 | -35.7 |
| 25.5 | 20 | -24.4 | -31.7 | -37.8 | -34.3 |
