# Supplementary material for: Carbon Availability Modifies Temperature Responses of Heterotrophic Microbial Respiration, Carbon Uptake Affinity, and Stable Carbon Isotope Discrimination
Source: Front Microbiol. 2016 Dec 26;7:2083. doi: 10.3389/fmicb.2016.02083 (PMC5184216; doi:10.3389/fmicb.2016.02083)
Supplement: Supplementary file 2 [file Table_2.docx]

Supplementary Table 2. Model parameters for microbial specific respiration rate, affinity, and δ^13^C of microbial biomass and CO_2_, assessed using *P. fluorescens* grown in chemostats with varying temperature and cellobiose concentrations.

|  | | | Slope ± 1SE | Intercept ± 1SE |
| --- | --- | --- | --- | --- |
| Specific respiration rate (mg C g^-1^ C h^-1^ °C^-1^) | | | | |
|  | 1 mM cellobiose | 16.16 ± 1.78 (**p<0.001**) | | -160.31 ± 31.60 (**p=0.004**) |
|  | 20 mM cellobiose | 2.06 ± 0.84 (p=0.071) | | 36.94 ± 15.03 (p=0.069) |
| C uptake affinity (mL mg^-1^ C h^-1^ °C^-1^) | | | | |
|  | 1 mM cellobiose | | 0.21 ± 0.02 (**p<0.001**) | -1.10 ± 0.33 (**p=0.022**) |
|  | 20 mM cellobiose | | 0.00 ± 0.00 (p=0.177) | 0.06 ± 0.01 (**p=0.002**) |
| δ^13^C of microbial biomass (‰°C^-1^) | | | | |
|  | 1 mM cellobiose | | 0.23 ± 0.08 (**p<0.007**) | -39.87 ± 2.65 (**p<0.001**) |
|  | 20 mM cellobiose | | 0.23 ± 0.08 (**p<0.007**) | -35.60 ± 1.67 (**p<0.001**) |
| δ^13^C of respired CO_2_ (‰°C^-1^) | | | | |
|  | 1 mM cellobiose | | 0.23 ± 0.08 (**p<0.007**) | -47.13 ± 2.33 (**p<0.001**) |
|  | 20 mM cellobiose | | 0.23 ± 0.08 (**p<0.007**) | -45.29 ± 4.36 (**p<0.001**) |

Significant parameters were highlighted in bold.
